# Identification of key genes CCL5, PLG, LOX and C3 in clear cell renal cell carcinoma through integrated bioinformatics analysis

**DOI:** 10.3389/fmolb.2025.1587196

**Published:** 2025-05-06

**Authors:** Zhenwei Xie, Cheng Feng, Yude Hong, Libo Chen, Mingyong Li, Weiming Deng

**Affiliations:** ^1^ Department of Kidney Transplantation, The Third Affiliated Hospital of Sun Yat-sen University, Guangzhou, China; ^2^ Department of Thyroid and Galactophore Surgery, People’s Hospital of Longhua, Shenzhen, China; ^3^ Department of Urology, The Third Affiliated Hospital of Sun Yat-sen University, Guangzhou, China; ^4^ Department of Urology, The First Affiliated Hospital, Hengyang Medical School, University of South China, Hengyang, China

**Keywords:** ccRCC, integrated bioinformatics analysis, hub genes, prognosis, biomarkers

## Abstract

**Background:**

Clear Cell Renal Cell Carcinoma (ccRCC) is a malignant tumor with high mortality and recurrence rates and the molecular mechanism of ccRCC genesis remains unclear. In this study, we identified several key genes associated with the prognosis of ccRCC by using integrated bioinformatics.

**Methods:**

Two ccRCC expression profiles were downloaded from Gene Expression Omnibus and one dataset was gained from The Cancer Genome Atlas The Robust Rank Aggregation method was used to analyze the three datasets to gain integrated differentially expressed genes The Gene Ontology and KEGG analysis were performed to explore the potential functions of DEGs. The Search Tool for the Retreival of Interacting Genes/Proteins (STRING) and Cytoscape software were used to construct protein-protein interaction network and module analyses to screen the hub genes. Spearman’s correlation analysis was conducted to evaluate the interrelationships among the hub genes. The prognostic value was evaluated through K-M survival analysis, Cox regression analysis, and receiver operating characteristic curve analysis to determine their potential as prognostic biomarkers in ccRCC. The expression of hub genes between ccRCC and adjacent normal tissues was analyzed by RT-qPCR, Western blotting, and immunohistochemical (IHC).

**Result:**

125 DEGs were identified using the limma package and RRA method, including 62 up-expressed genes and 63 down-expressed genes. GO and KEGG analysis showed some associated pathways. Spearman’s correlation analysis revealed that the hub genes are not only interrelated but also closely associated with immune cell infiltration. Gene expression analysis of the hub genes based on the TCGA-KIRC cohort, along with K-M survival analysis, Cox regression, and ROC curve analysis, consistently demonstrated that CCL5, LOX, and C3 are significantly upregulated in ccRCC and are associated with poor clinical outcomes. In contrast, PLG showed opposite result. These results were further validated at the mRNA and protein levels.

**Conclusion:**

Our findings indicate that CCL5, LOX, C3, and PLG are significantly associated with the progression and prognosis of ccRCC, highlighting their potential as prognostic biomarkers. These results provide a foundation for future research aimed at uncovering the underlying mechanisms and identifying potential therapeutic targets for ccRCC.

## Introduction

Renal cell carcinoma (RCC), a type of urologic neoplasm, originates from renal tubular epithelial cells. RCC includes several subvariants, with clear cell RCC (ccRCC) accounting for approximately 80% of cases (Siegel et al., 2024; Young et al., 2024). The 5-year survival rate for localized ccRCC exceeds 90%, whereas metastatic disease is associated with a dismal 12% survival rate. ccRCC exhibits intrinsic resistance to conventional chemoradiotherapy. New treatment options for patients with metastatic ccRCC include immune checkpoint inhibitors and tyrosine kinase inhibitors. In recent time, surgical resection is still the primary therapeutic option (Rose and Kim, 2024). Despite curative intent in early-stage disease, recurrent ccRCC remains a clinical challenge due to limited therapeutic options (Brugarolas, 2014). Underscoring the need for novel therapeutic strategies. Numerous studies have focused on identifying genes associated with the development and prognosis of ccRCC. It has been shown that a loss-of-function mutation in the von Hippel–Lindau (VHL) gene promotes the occurrence and development of ccRCC (Choueiri and Kaelin, 2020). With the rapid advancement of microarray and high-throughput sequencing technologies, an increasing number of researchers are leveraging these methods to identify novel biomarkers that could improve the prognosis of ccRCC patients. For instance, Dopamine transporter SLC6A3 has been identified as a novel biomarker with diagnostic potential in ccRCC patients (Schrödter et al., 2016). In another study, CXCL13 demonstrated strong diagnostic and prognostic value and may serve as a potential biomarker and therapeutic target for ccRCC (Xu et al., 2019). Despite the identification of several biomarkers, such as VHL, SLC6A3, and CXCL13, their clinical application remains limited due to insufficient specificity and reproducibility across different patient cohorts. Many potential biomarkers have been identified through transcriptomic studies; however, their roles in prognosis prediction and therapeutic targeting are not well established. Given these limitations, our study employs integrated bioinformatics analysis of multiple datasets combined with clinical ccRCC tissue validation to identify novel hub genes with significant prognostic value, potentially addressing gaps in ccRCC biomarker research and facilitating clinical translation.

In the current study, gene expression profile datasets were downloaded from the GEO database and TCGA-KIRC, and further analyzed using R software. Due to heterogeneity in experimental samples, the results obtained from different platforms were inconsistent, the Robust Rank Aggregation (RRA) approach, specifically designed to compare multiple ranked gene lists, was used to integrate the data and gain integrated differentially expressed genes (DEGs). Functional enrichment analyses, including Gene Ontology (GO), Kyoto Encyclopedia of Genes and Genomes (KEGG), were performed using the DAVID database to elucidate the biological roles of DEGs. The Search Tool for the Retrieval of Interacting Genes/Proteins (STRING) database was utilized to construct the protein-protein interaction (PPI) network of the DEGs. After identifying the hub genes, modules from the PPI network were constructed. Finally, K-M plotting, Cox regression analysis, and ROC curve analysis are commonly employed to evaluate the prognostic significance of hub genes and to identify their potential as prognostic biomarkers in ccRCC. To further validate the reliability of the experimental findings, the expression levels of four genes (CCL5, PLG, LOX, and C3) were examined in paired ccRCC and adjacent normal tissues using RT-qPCR, Western blotting, and IHC assays.

In this study, a multidimensional bioinformatics approach was employed to systematically investigate the differential expression and functional enrichment of four candidate genes using data from multiple public databases. Our findings underscore the potential of these genes as novel biomarkers and therapeutic targets in ccRCC.

## Materials and methods

### Data acquisition

Datasets (GSE40435 and GSE53757) were downloaded from the GEO database (http://www.ncbi.nlm.nih.gov/geo/). The TCGA-KIRC data, which contained 539 ccRCC tumor tissue samples and 72 normal kidney tissue samples, was downloaded from TCGA (https://www.cancer.gov/aboutnci/organization/ccg/research/structural-genomics/tcga). The GSE40435 dataset (GPL10558 Illumina HumanHT-12 V4.0 expression BeadChip) contains 101 pairs of ccRCC tumors and adjacent non-tumor renal tissue. The Platform of the GSE53757 dataset is the GPL570 [HG-U133_Plus_2] Affymetrix Human Genome U133 Plus 2.0 Array, and it consists of 72 ccRCC samples and 72 matched normal tissue samples. Then, normalization of each dataset was performed by using normalize Between Arrays function in the limma R package (http://www.bioconductor.org/). All gene expression data were log2-transformed.

### Identification of DEGs

The limma R software package was utilized to identify the DEGs in GSE40435 and GSE53757, and the TCGA-KIRC datasets were screened for DEGs by using the edgeR package. The quantile normalization was carried out to normalize the data, then the genes with the threshold of |logFC| > 2 and P < 0.05 were considered statistically significant DEGs. Upregulated and downregulated genes were screened by using the Robust Rank Aggregation (RRA) analysis (http://cran.r-project.org/). The integrated DEGs of three datasets were exhibited by volcano map and hierarchical clustering heat map.

### Functional enrichment analysis

The Database for Annotation, Visualization, and Integrated Discovery (DAVID) (http://david.ncifcrf.gov/home.jsp; version 6.8) is a generally used database for gene enrichment and functional annotation analyses. The GO analysis is extensively used in bioinformatics, including biological process (BP), cellular component (CC), and molecular function (MF). The KEGG is an online database which includes most known metabolic pathway maps and regulatory pathway maps.

### PPI network construction

The online database STRING (http://string-db.org) and Cytoscape software (Version 3.6.6, http//www.cytoscape.org/) were utilized to perform PPI network of DEGs and identify the hub genes, with confidence score threshold of 0.4 (medium confidence). Besides, the networks of highly interconnected nodes from the PPI network were analyzed by using the Cytoscape software with the plug-in of Molecular Complex Detection (MCODE). Top 10 Hub genes were visualized in Cytoscape software by using the cytoHubba plug-in.

### Immune cell infiltration analysis by ssGSEA

Single-sample Gene Set Enrichment Analysis (ssGSEA) was performed to evaluate the infiltration levels of 24 immune cell types in ccRCC samples from TGCA-KIRC. Spearman’s correlation analysis was then conducted to assess the associations between the expression levels of CCL5, PLG, LOX, and C3 and the relative abundance of these immune cell populations.

### Survival analysis of hub genes

Survival curves were generated using the Kaplan-Meier Plotter online tool (http://kmplot.com/analysis/). The optimal cut-off value for gene expression stratification was automatically determined by evaluating all possible thresholds between the lower and upper quartiles. The threshold with the highest statistical significance (lowest FDR, corrected by the Benjamini-Hochberg method) was selected. In cases of multiple equally significant cut-offs, the one with the highest hazard ratio (HR) was chosen for final analysis. Mann-Whitney U test analyzed the expression level of hub genes in the three data sets, and the ComplexHeatmap package was used to visualize the expression level of hub genes in TCGA-KIRC. To further personalize the prediction of overall survival at 1, 3, and 5 years, a nomogram was constructed based on the results of multivariate Cox regression analysis. The rms R package was utilized to generate the nomogram, which incorporated the expression levels of the identified hub genes. Additionally, the predictive performance of the nomogram was compared with that of individual prognostic factors using the concordance index (C-index) and receiver operating characteristic (ROC) curve analysis.

### RNA extraction and RT-qPCR

In this study, 12 matched ccRCC tissues and adjacent normal tissues were collected from the First Affiliated Hospital of the University of South China (Hengyang, China) between January 2024 and June 2024. All the human tissue protocols were strictly conducted in accordance with the Declaration of Helsinki and were approved by the Biomedical Ethics Committee of the University of South China (IRB-2024-019). Total RNA was extracted from ccRCC and adjacent normal tissues using TRIzol reagent (Thermo Fisher Scientific, United States) and transcribed to cDNA using HiScript III RT SuperMix + gDNA Wiper Kit (Vazyme, Nanjing, China). Real-time PCR analysis was performed using the ABI PRISM 7500 (Thermo Fisher Scientific, Inc.) with SYBR Green qPCR Master Mix (Vazyme, Nanjing, China). CCL5, LOX, C3, PLG, and GAPDH sequences were synthesized by IGE Biotech (Guangzhou, China). And are shown in Supplementary Table S1. The relative expression was calculated using the 2^−ΔΔCT^ method, with normalization to the GAPDH gene.

### Western blotting

To validate the differential expression of the hub genes at the protein level, Western blotting was performed on tissue samples obtained from clear cell renal cell carcinoma (ccRCC) patients. Fresh tumor tissues and matched adjacent normal renal tissues were collected immediately after surgical resection and stored at −80°C until use. ccRCC tissues and adjacent normal tissues were lysed using ice-cold lysis buffer containing 0.25 M NaCl, 50 mM Tris-HCl (pH 7.4), 0.5% NP-40, 1 mM EDTA, 1 mM Na_3_VO_4_, 1 mM NaF, 1% protease inhibitor cocktail, and 1 mM PMSF. Total protein concentrations were measured using the BCA Protein Assay Kit (Thermo Fisher Scientific, United States). Equal amounts of protein were separated by SDS-PAGE and transferred onto polyvinylidene difluoride (PVDF) membranes (Millipore, United States).

The membranes were blocked with 5% fat-free milk in PBST (PBS with 0.1% Tween-20) for 2 h at room temperature, followed by overnight incubation at 4°C with primary antibodies. After three washes with PBST, the membranes were incubated with horseradish peroxidase (HRP)-conjugated secondary antibodies at room temperature for 1 h. Immunoreactive bands were visualized using enhanced chemiluminescence (ECL) substrate (sc-2048, Santa Cruz Biotechnology) and imaged using a chemiluminescence detection system.

Primary antibodies used included: CCL5 (ab307712, 1:1,000, Abcam), LOX (#58135, 1:1,000, Cell Signaling Technology), C3 (#97425, 1:1,000, Cell Signaling Technology), and PLG (ab242329, 1:1,000, Abcam). Tubulin (TU-01, 1:2,500, Thermo Fisher Scientific) was used as a loading control. Band intensities were quantified using ImageJ software, and the relative expression levels of target proteins were normalized to Tubulin.

### Immunohistochemistry (IHC)

Six matched ccRCC tissues and adjacent normal tissues were paraffin-embedded and then cut into 4-μm slides for IHC staining. Briefly, the sections were processed with deparaffinized using xylene and ethanol, and then added EDTA buffer for antigen repair. After being blocked with 5% goat serum for 2 h, the slides were incubated with the primary antibody against CCL5(CST, #36467), PLG (Abcam, ab242329), LOX (Abcam, ab221936), C3 (Abcam, ab11871) overnight at 4°C, followed by secondary antibodies for 1 h at room temperature. Positive staining was analyzed by two experienced tumor pathologists using ImageJ software to compare the differences between ccRCC tissue and adjacent normal tissue.

### Statistical analysis

Statistical analysis was performed using GraphPad Prism 8.0 (GraphPad Software, Inc., San Diego, CA, United States). R software version 3.5.0. was used for analysis. Correlations were determined by Spearman’s analysis. The mRNA and protein expression levels of the four hub genes between ccRCC and normal tissues were analyzed by Student’s t-test. P < 0.05 was considered statistically significant.

## Results

### Microarray data normalization and screening for DEGs in ccRCC

**FIGURE 1:**
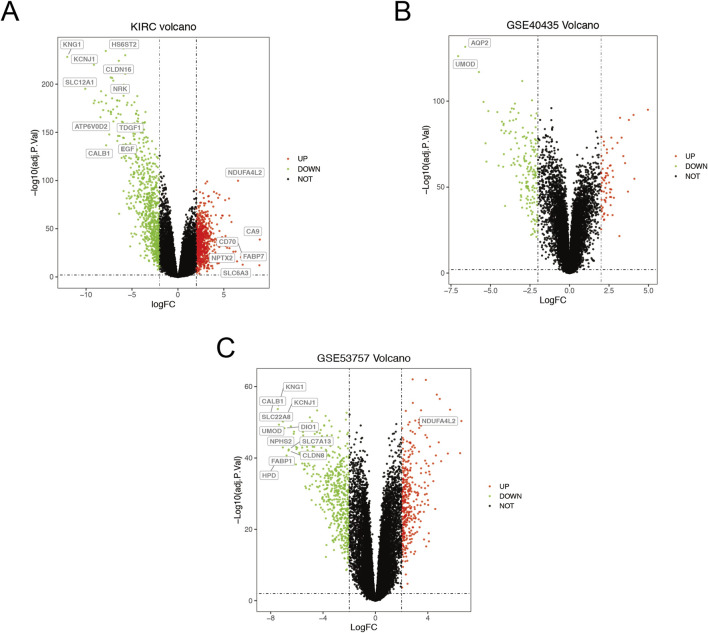
Volcano plot of differential data expressions between tumor and normal tissues. **(A)** TCGA-KIRC. **(B)** GSE43435. **(C)** GSE53757. The red points represent up-expressed genes, the green dots represent down-expressed genes (P < 0.05, |logFC|≥2). The black points represent undifferentiated genes. The genes listed in the box represent key genes (P < 0.001, |logFC|>6.5).

**FIGURE 2:**
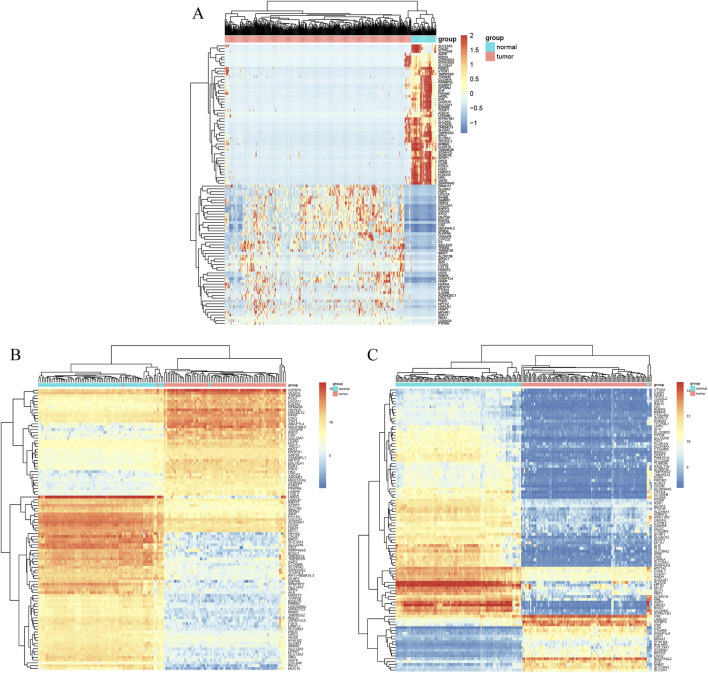
Cluster heat map of the top 100 DEGs. **(A)** TCGA-KIRC. **(B)** GSE40435. **(C)** GSE53757. Data were shown as the heatmap. Red grid suggests that the gene expression is up-expressed, green grids shows that down-expressed, black grid shows that no significant difference exists.

**FIGURE 3:**
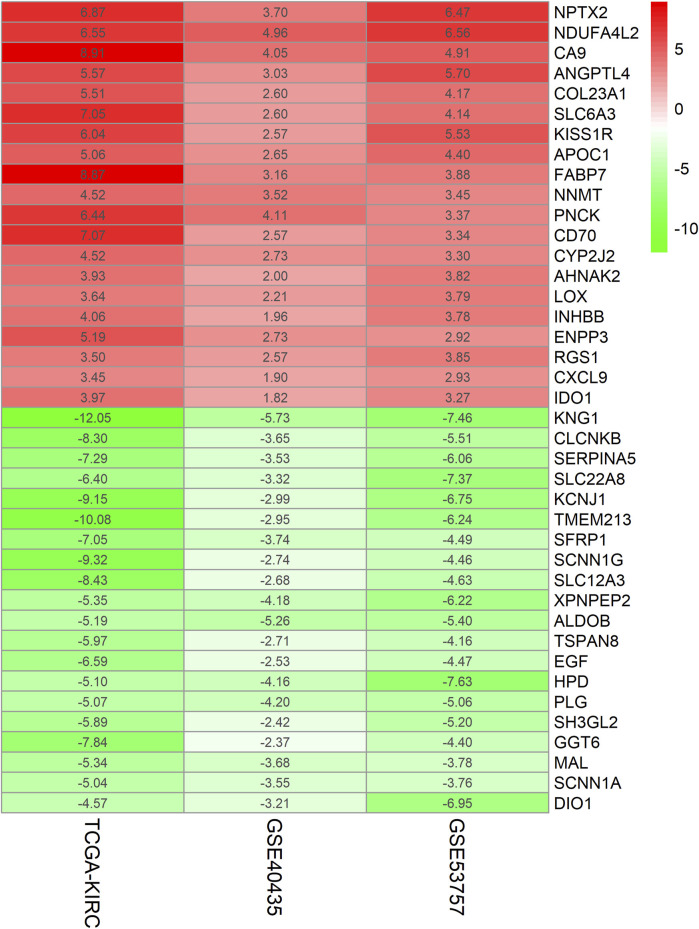
Heatmap of Top40 gene expression microarray. The abscissa represents the GEO ID, the ordinate represents the gene name, and the red represents upregulated in ccRCC compared with normal tissues. The green represents downregulated in ccRCC compared with normal tissues, the value represents the log FC value.

The ccRCC chip expression datasets (GSE40435 and GSE53757) were downloaded from the GEO and were normalized by using the limma R package, and the charts were displayed in Supplementary Figure S1. Which also presents the normalization of gene expression profiles across different datasets, ensuring data comparability before differential expression analysis. The DEGs were identified with the screening criteria (P < 0.05 and |logFC|≥2), the GSE40435 dataset was screened out 236 differential genes, among which 71 were upregulated and 165 were downregulated. The GSE53757 dataset screened out 886 differential genes, among which 365 were upregulated and 521 were downregulated. In addition, the author obtained 755 upregulated genes and 773 downregulated genes by analyzing gene expression data obtained from the TCGA-KIRC dataset. The DEGs from the three datasets were presented respectively by volcano plot (Figure 1). The cluster heatmaps present the top 100 upregulated and 100 downregulated genes (Figure 2). Then, the above microarray databases of ccRCC from GEO database were obtained by using the limma R package, and the TCGA-KIRC database was analyzed and classified by the edgeR package. The common DEGs were screened by the RRA package (P < 0.05, |logFC| ≥ 2). As a result, 125 integrated DEGs were identified, consisting of 62 up-expressed genes and 63 down-expressed genes. The heatmap presents the top 20 upregulated and 20 downregulated genes (Figure 3).

### Go and KEGG analysis of DEGs

**FIGURE 4:**
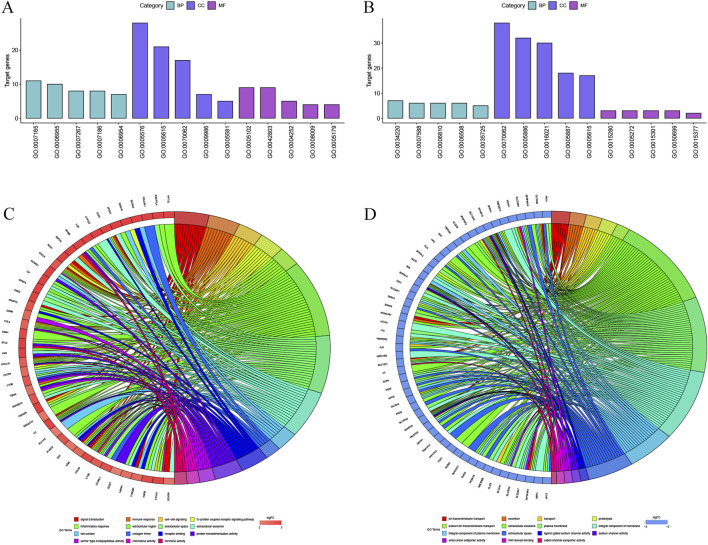
The DEGs enriched GO terms **(A)** Upregulated DEGs with the top 15 enriched terms. **(B)** Downregulated DEGs with the top 15 enriched terms. **(C)** The contribution of upregulated DEGs in KIRC to different GO enrichment pathways. **(D)** The contribution of downregulated DEGs in KIRC to different GO enrichment pathways.

The author then performed a Gene Ontology analysis of the hub genes using the DAVID Functional Annotation tool. Significant results of GO term analysis included BP, CC, and MF groups, respectively. As displayed in Figure 4, the upregulated DEGs were mainly enriched in signal transduction, immune response, cell-cell signaling, G-protein coupled receptor signaling pathway, and inflammatory response under BP, while downregulated genes were mainly enriched in ion transmembrane transport, excretion, transport, proteolysis, and sodium ion transmembrane transport. The upregulated genes were primarily enriched in the extracellular region, extracellular space, extracellular exosome, cell surface, and collagen trimer, while the downregulated genes were chiefly enriched in the extracellular exosome, plasma membrane, integral component of membrane, integral component of plasma membrane, and extracellular space of CC. The upregulated DEGs were mainly enriched in receptor binding, protein homodimerization activity, serine-type endopeptidase activity, chemokine activity, and hormone activity; however, the downregulated DEGs were mainly enriched in ligand-gated sodium channel activity, sodium channel activity, anion: anion antiporter activity, WW domain binding, and cation: chloride symporter activity at MF.

**FIGURE 5:**
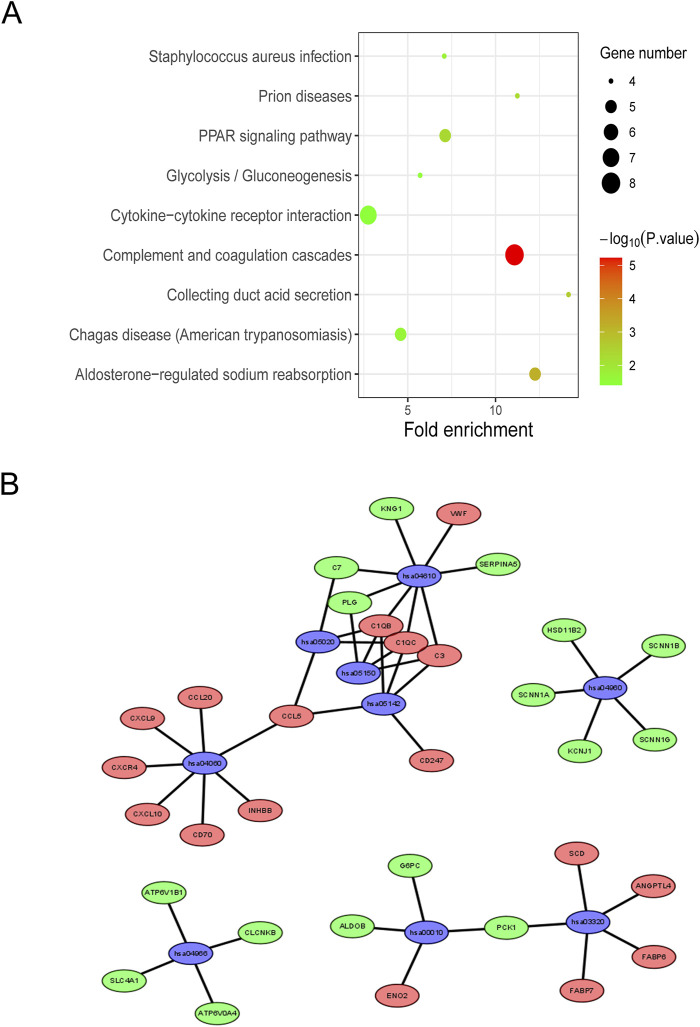
KEGG enrichment analysis of integrated DEGs **(A)** KEGG pathway enrichment analysis of integrated DEGs. **(B)** Network map of KEGG enriched pathways, blue represents the pathways, red represents the genes that are upregulated, and green represents the downregulated genes.

The KEGG pathway enrichment analysis was performed to screen the potential pathways of the integrated DEGs using the DAVID database. As shown in Figure 5A, the DEGs were enriched in nine pathways, which mainly had relevance to Cytokine-cytokine receptor interaction and other pathways. The network diagram was generated using Cytoscape software, which is shown in Figure 5B. Previous research has demonstrated that CCL5 promotes hepatic inflammation and carcinogenesis through activation of the AKT/HIF1α signaling pathway (Zong et al., 2023). Another study has revealed that tumor-associated macrophage-derived CCL5 promotes ccRCC progression and fosters an immunosuppressive tumor microenvironment through modulation of the PI3K/AKT signaling pathway (Xu et al., 2022), underscoring the crucial involvement of cytokine-cytokine receptor interactions in tumorigenic processes.

### Construction of PPI network and screening of hub genes

**FIGURE 6:**
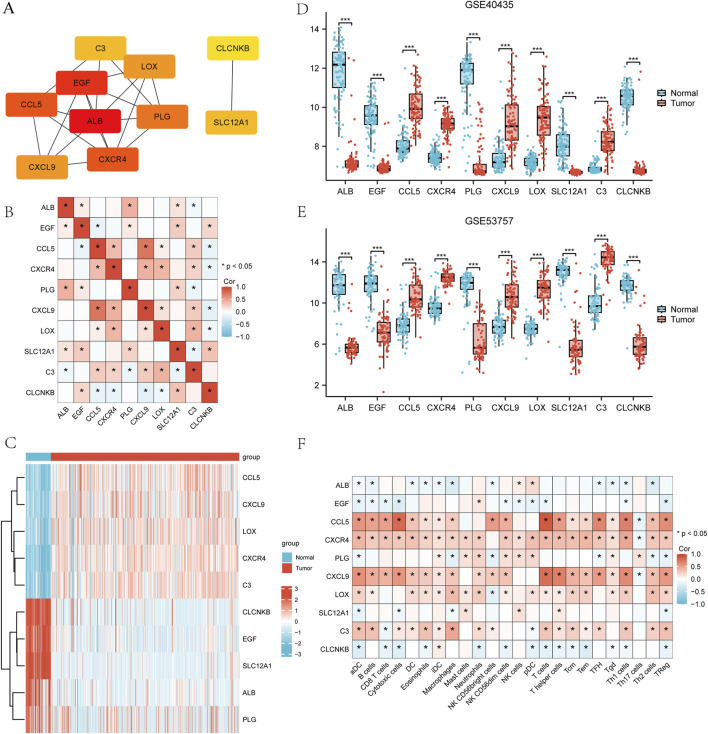
Construction of PPI network and screening of hub genes **(A)** Top 10 hub genes filtered by cytoHubba plug-in. **(B)** Spearman’s correlation analysis illustrating the correlations among the hub genes in the TCGA-KIRC cohort. **(C–E)** Expression levels of hub genes in the TCGA-KIRC data, GSE40435 dataset, and GSE53757 dataset. **(F)** Spearman’s correlation analysis between hub genes and 24 types of immune cells in the TCGA-KIRC cohort.

The STRING online database was utilized to analyze 125 integrated DEGs and to construct PPI network. Supplementary Figure S2 illustrates the detailed interactions of hub genes in the PPI network, highlighting the most interconnected node. The following 10 hub genes were screened by Cytoscape software and results are shown in Figure 6A, which are ALB, EGF, CCL5, CXCR4, PLG, LOX, C3, CXCL9, SLC12A1, and CLCNKB. Spearman’s correlation analysis was performed to evaluate the interrelationships among the hub genes in the TCGA-KIRC cohort. The results were visualized as a heatmap using the ggplot2 package in R. As illustrated in the heatmap, significant correlations were observed among the hub genes, suggesting that they may exert a synergistic effect during the progression of ccRCC (Figure 6B). Subsequently, the author verified the expression levels of the identified hub genes across two GSEA datasets and TCGA-KIRC. As shown in Figures 6C–E, these hub genes exhibited consistent patterns of upregulation or downregulation in all three datasets. Notably, the direction of expression changes for each hub gene remained concordant across datasets, indicating the robustness and reliability of our findings. The correlations between hub genes and 24 types of immune cell (aDC [activated DC]; B cells; CD8 T cells; Cytotoxic cells; DC; Eosinophils; iDC [immature DC]; Macrophages; Mast cells; Neutrophils; NK CD56bright cells; NK CD56dim cells; NK cells; pDC [Plasmacytoid DC]; T cells; T helper cells; Tcm [T central memory]; Tem [T effector memory]; TFH [T follicular helper]; Tgd [T gamma delta]; Th1 cells; Th17 cells; Th2 cells; T Reg) (Bindea et al., 2013) were analyzed using Spearman’s correlation in the TCGA-KIRC cohort. The results were visualized as heatmaps using the ggplot2 package in R. As shown in Figure 6F, the expression levels of several hub genes were significantly positively correlated with immune cell infiltration (p < 0.05). These findings suggest that the hub genes may be involved in modulating the tumor immune microenvironment and play important roles in immune infiltration in ccRCC.

### Prognostic significance and expression of hub genes

**FIGURE 7:**
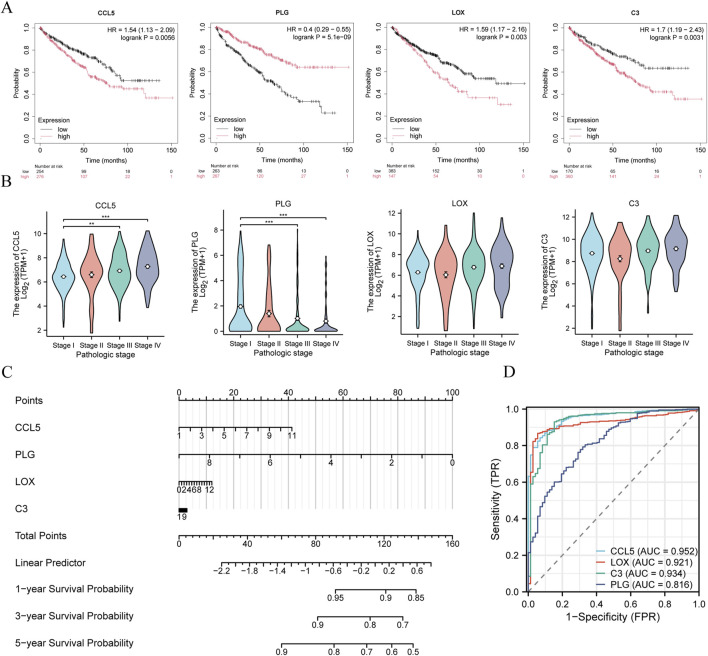
Prognostic significance and expression of hub genes **(A)** Kaplan–Meier survival curves illustrating the prognostic significance of the four hub genes in ccRCC patients. **(B)** Expression levels of the four hub genes across different pathological stages in the TCGA-KIRC dataset. **(C)** Nomogram predicting the 1-, 3-, and 5-year overall survival probabilities for ccRCC patients based on the expression levels of the four hub genes. **(D)** ROC curve analysis demonstrating the discriminatory power of the four hub genes in distinguishing tumor from adjacent normal tissues in the TCGA-KIRC cohort.

The Overall Survival (OS) of the above 10 genes in ccRCC was analyzed using the K-M plotter online tool. Our analysis revealed significant OS associations for CCL5, PLG, LOX, CXCR4, and C3 among the identified hub genes. Of these, CXCR4 has gained particular attention in recent clinical research for its dual diagnostic and prognostic value in ccRCC (Alsayed et al., 2022). The oncogenic function of CXCR4 exhibits tissue-specific regulation: while DUXAP8 lncRNA upregulates CXCR4 *via* miR-223 sequestration in papillary thyroid carcinoma (Liu et al., 2021), miR-1246-mediated CXCR4 suppression attenuates proliferative and migratory capacities in renal cell carcinoma (Liu and Fan, 2020). The remaining five genes did not exhibit statistically significant OS differences and were not prioritized for further investigation. So, we focused on discussing the role of CCL5, PLG, LOX, and C3 in the progression of ccRCC. As shown in Figure 7A, ccRCC patients with higher expression of CCL5, LOX, and C3 had poorer OS, while patients with higher expression of PLG had better OS. Subsequently, we investigated the association between the expression of CCL5, PLG, LOX, and C3 and the pathological stage of ccRCC. As shown in Figure 7B, TCGA-KIRC samples were stratified into four groups based on clinical and pathological staging: stage I (n = 276), stage II (n = 59), stage III (n = 125), and stage IV (n = 83). Differential expression analysis revealed that CCL5 and PLG were significantly upregulated in stages III and IV compared to stage I (p < 0.05), suggesting their potential roles in promoting disease progression. In contrast, the expression levels of LOX and C3 did not show significant differences across pathological stages. Statistical significance was assessed using the Kruskal-Wallis test, followed by Dunn’s test for multiple comparisons. To further elucidate the relationship between these genes and OS, a nomogram was constructed. In this nomogram, a point scale was used to assign scores based on the expression levels of the selected genes. For each gene, a vertical line was drawn upward to determine its corresponding point value (Liu et al., 2018). The total points, calculated as the sum of points, was then rescaled to a range from 0 to 160. To estimate the probability of overall survival at 1, 3, and 5 years, a vertical line was drawn downward from the total points axis to the survival probability axis. As shown in Figure 7C, higher expression levels of CCL5, LOX, and C3 were associated with higher total points and correspondingly lower 5-year survival probabilities. Conversely, lower expression of PLG contributed to higher total points and a worse predicted prognosis. These findings are consistent with the OS trends observed in Figure 7A, further supporting the prognostic value of these genes in ccRCC. Moreover, receiver operating characteristic (ROC) curve analysis was performed to evaluate the diagnostic efficacy of CCL5, PLG, LOX, and C3 in distinguishing ccRCC from normal tissues. The results demonstrated that all four genes exhibited strong discriminatory power, with area under the curve (AUC) values of 0.952 for CCL5, 0.816 for PLG, 0.921 for LOX, and 0.934 for C3, respectively (Figure 7D). These findings suggest that the expression levels of these genes may serve as potential diagnostic biomarkers for ccRCC.

**FIGURE 8:**
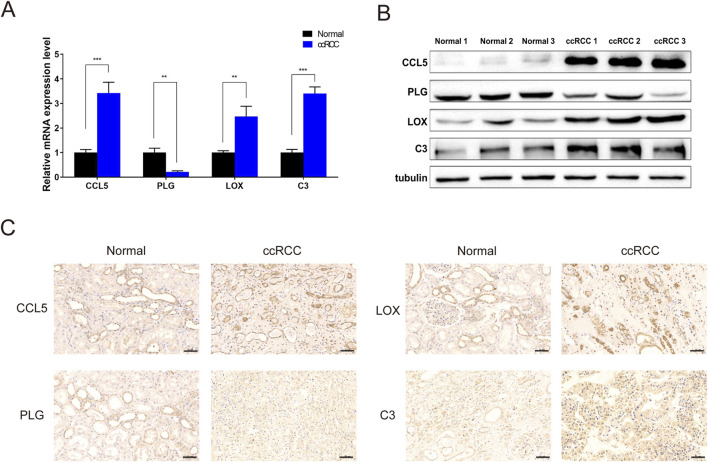
The expression of four genes between ccRCC and adjacent normal tissue **(A)** qPCR for mRNA expression levels. **(B)** Western blot analysis showing differential expression of CCL5, PLG, LOX, and C3 between ccRCC tissues and matched adjacent normal kidney tissues. **(C)** Immunohistochemical detection for protein expression levels of CCL5, PLG, LOX, and C3. ∗∗P < 0.01, ∗∗∗P < 0.001.

To validate the results of the previous analysis, the mRNA and protein expression of the four hub genes were analyzed in normal renal tissues and ccRCC with qRT-PCR, Western blotting and immunohistochemistry. In agreement with the online database, CCL5, C3, and LOX were upregulated, whereas PLG was downregulated in the ccRCC relative to normal tissues (Figures 8A–C).

## Discussion

Traditional chemotherapy and radiotherapy are rarely effective in the treatment of ccRCC, and partial or total surgical resection of the kidney is the main treatment, but there is still a high risk of recurrence after surgery (Lih et al., 2023). Recently, more and more researchers have tried to find prognostic biomarkers for ccRCC with the intention to identify patients who were at higher risk of recurrence and death (Petitprez et al., 2021). With the rapidly development of microarrays and high-throughput sequencing, numerous underlying molecular targets for ccRCC were extensively explored. For example, EZH2, SLC6A3 and SPTLC1 were identified as potential prognosis-related genes in ccRCC (Schrödter et al., 2016; Ho et al., 2017; Zhu et al., 2020). More possible biomarkers of ccRCC need to be explored for purpose of improving patient prognosis. To this end, we analyzed the GSE40435 and GSE53757 datasets and TCGA-KIRC datasets using the RRA method, and 125 integrated DEGs were found. These genes were then subjected to GO and KEGG analysis. The results showed that the upregulated genes were mainly enriched in signal transduction (ontology: BP), the extracellular region (ontology: CC) and receptor binding (ontology: MF), and downregulated genes were mainly enriched in ion transmembrane transport (ontology: BP), extracellular exosomes (ontology: CC) and ligand-gated sodium channel activity (ontology: MF). The results of KEGG analysis indicated that these integrated DEGs are mostly enriched in these pathways: Complement and coagulation cascades, Aldosterone-regulated sodium reabsorption, Cytokine-cytokine receptor interaction and PPAR signaling pathway. The PPI network was constructed by using STRING online database to analyze 125 integrated DEGs, and further verification based on the cytoscape software indicated the following 10 hub genes (ALB, EGF, CCL5, CXCR4, PLG, LOX, C3, CXCL9, SLC12A1, and CLCNKB) had significantly highly expressed. Then, through the K-M plotter online tool, the author proved that ccRCC patients with high CCL5, LOX and C3 expression had a poor prognosis, while ccRCC patients with high PLG expression had a better prognosis. CXCR4 has been established as a critical mediator in ccRCC progression, contributing to tumor growth and metastasis through multiple signaling pathways. Although the other five hub genes did not show significant prognostic value in OS analysis, their biological roles in ccRCC progression warrant further investigation. Future studies may explore their potential involvement in tumorigenesis and therapeutic responses. Subsequently, a nomogram and ROC curve analysis were constructed to further evaluate the prognostic value of these genes in ccRCC. Finally, RT-qPCR, Western blotting, and IHC assays were performed to validate the differential expression of the hub genes between ccRCC tissues and adjacent normal renal tissues. The results were consistent with our expectations: CCL5, LOX, and C3 were significantly upregulated in tumor tissues, whereas PLG exhibited markedly higher expression in normal tissues.

CCL5 belongs to the CC chemokine superfamily and has a high affinity to interact with its receptor (Bronte and Bria, 2016). CCL5 is involved in the occurrence, development and metastasis of various cancers (Tian et al., 2022). It can contribute to the deterioration of prognosis in breast cancer patients by interacting with CCR3 and can also interact with CCR5 to affect the progression and metastasis of hepatocellular carcinoma (Singh et al., 2020; Yamaguchi et al., 2021). Tumor-associated macrophages (TAMs) dominate the evolution and outcomes of cancers by perturbing the tumor microenvironment (TME) (Galon and Bruni, 2020). In ccRCC specimens exhibiting CCL5 upregulation, a significant increase was observed in both CCL5+ tumor-associated macrophages (TAMs) and PD-L1+ CD68^+^ TAM populations, indicative of an immunosuppressive tumor immune microenvironment (TIME). Additionally, CCL5 promotes epithelial-mesenchymal transition (EMT) *via* the PI3K/AKT pathway, contributing to tumor progression and immune evasion. Additionally, CCL5+ tumor-associated macrophages infiltration correlates with poor prognosis, highlighting its clinical significance (Xu et al., 2022). Another study demonstrated that an 8-hydroxyquinoline derivative effectively suppresses esophageal tumor invasion by downregulating CCL5 expression, suggesting that CCL5 may serve as a potential therapeutic target for inhibiting tumor progression (Tang et al., 2024). In ccRCC studies, CCL5 was found to be highly expressed in ccRCC patients with a poor prognosis. CCL5 mediates the formation of TAMs, which in turn promotes target drug resistance and progression in ccRCC (Wang et al., 2021).

Lysyl oxidase (LOX), as a member of lysyl oxidase family, is an extracellular copper-dependent amine oxidase which plays a vital role in fibrogenesis, extracellular matrix remodeling, and tumorigenesis (Ye et al., 2020). LOX plays a crucial role in extracellular matrix (ECM) remodeling by facilitating collagen cross-linking. This process contributes to angiogenesis and tumor microenvironment (TME) modulation, which are critical for tumor progression. Recent studies highlight the significant involvement of LOX in liver cancer, suggesting their potential as therapeutic targets in tumor (Lin et al., 2020). LOX catalyzes the cross-linking of elastin and collagen in the extracellular matrix (ECM) and promotes the formation of tumor cell migration and metastasis (Ramos et al., 2022). In several integrative bioinformatics studies, LOX showed prominent predictive value. LOX expression was associated with lymph node metastasis, distant tumor metastasis, and poor prognosis in GC patients, and the integrated analysis supported LOX as a specific diagnostic and prognostic biomarker for GC patients (Jia et al., 2022). In addition, LOX has an excellent predictive value for chemotherapy, immunotherapy, and prognosis in patients with glioma (Xia et al., 2022). A recent study demonstrated that LOX inhibition reduces stromal matrix deposition, thereby enhancing chemotherapy efficacy in pancreatic ductal adenocarcinoma. These findings highlight LOX as a potential therapeutic target for clinical intervention (Chitty et al., 2023). Few studies are available concerning LOX in ccRCC. One study found that endogenous LOX is overexpressed in ccRCC, participates in a positive-regulative loop with HIF-1α, and contributes to ccRCC progression by increasing cell adhesion, migration, and collagen matrix stiffness (Di Stefano et al., 2016). Another study found that high expression of LOX was associated with decreased overall and metastasis-free survival in ccRCC (Añazco et al., 2021). The previous studies have limitations, and more in-depth studies are needed to confirm that LOX can be used as a biomarker and pharmacological target in ccRCC.

Complement component 3 (C3), as a protein-coding gene, participates in the genesis and development of numerous diseases, including C3 deficiency, autosomal recessive, and glomerulopathy (Assirelli et al., 2020; Smith et al., 2019). As part of the immune system, it is involved in the genesis and progression of multiple tumor types. The main pathways in which it is involved are the immune response lectin-induced complement pathway and signaling by the GPCR. In previous reports, which indicated that tumor cell-derived C3 contributes to an immunosuppressive tumor microenvironment (TME) by activating the C3a-C3aR-PI3Kγ signaling pathway in tumor-associated macrophages (TAMs), thereby repressing antitumor immunity. Additionally, C3 deletion in high-C3-expressing tumor cells has been shown to enhance the efficacy of anti–PD-L1 therapy, suggesting its potential as a therapeutic target (Zha et al., 2019). Overexpressed C3 could activate JAK2/STAT3 pathway, which has a correlation with the progression of gastric cancer (Yuan et al., 2020). In addition, C3 was proved to be a new immune marker for differentiating the prognosis of patients with colorectal adenocarcinoma (Liu and Wang, 2021). In a study investigating gastric cancer peritoneal metastases, blockade of the C3-C3AR1 axis was shown to disrupt stroma-myeloid crosstalk, leading to enhanced efficacy of immune checkpoint blockade (ICB) in preclinical models. These findings suggest that targeting C3 may represent a promising strategy to overcome stromal immunosuppression and potentiate ICB response in gastric cancer metastasis (Li et al., 2025).

Plasminogen (PLG) is the protein-encoding gene of plasminogen. Its protein coding product is a serine protease that exists as an inactive zymogen in blood plasma and it can be transformed into an active protease by activators such as tissue plasminogen activator (tPA) and urokinase plasminogen activator (uPA) (Syrovets et al., 2012). The interaction of PLG and TPA produces plasmin that promote fibrinolysis, an important process in cancer progression (Ranson and Andronicos, 2003). There is growing evidence that components of the plasminogen-plasma system are involved in tumor growth, invasion, and metastasis by modulating cell migration and angiogenesis (Didiasova et al., 2014; Heissig et al., 2021). Another study demonstrated that targeting the PLG receptor S100A10 suppresses angiogenesis in clear cell renal cell carcinoma (ccRCC), proving that PLG inhibition may represent a promising therapeutic strategy to impede tumor progression in ccRCC (Xiao et al., 2019). Our study suggests that PLG may be associated with the prognosis of ccRCC patients, but the underlying mechanism of this process is still obscure, further investigation needs to be performed to make out the concrete mechanism.

## Conclusion

In summary, this study aimed to elucidate the molecular mechanisms underlying ccRCC pathogenesis. By integrating bioinformatics analyses of GEO and TCGA-KIRC datasets, we identified four candidate genes—CCL5, PLG, LOX, and C3—with potential prognostic significance in ccRCC. Functional enrichment and survival analyses revealed that these genes are involved in critical biological processes, including immune regulation, extracellular matrix remodeling, and metabolic reprogramming, suggesting their roles in tumor progression and therapeutic resistance.

However, the precise molecular mechanisms through which these genes contribute to ccRCC progression remain to be fully elucidated. A limitation of the present study is its reliance on only three datasets; therefore, future research should incorporate larger clinical cohorts and additional sequencing data to enhance the robustness of these findings. Moreover, experimental validation through *in vitro* and *in vivo* studies will be essential to confirm the functional roles and therapeutic potential of these candidate biomarkers.

## Data Availability

The datasets presented in this study can be found in online repositories. The names of the repository/repositories and accession number(s) can be found below: https://www.ncbi.nlm.nih.gov/geo/, GSE40435 and GSE53757.
