# Supplementary material for: Identification of key genes CCL5, PLG, LOX and C3 in clear cell renal cell carcinoma through integrated bioinformatics analysis
Source: Front Mol Biosci. 2025 May 6;12:1587196. doi: 10.3389/fmolb.2025.1587196 (PMC12088980; doi:10.3389/fmolb.2025.1587196)
Supplement: Supplementary file 1 [file Supplementaryfile1.docx]

**Supplementary Table 1. The primers for RT-qPCR assay**

**Supplementary Table 2. DEGs (TCGA-KIRC, GSE40435, GSE53757)**

|  | **Gene Names** |
| --- | --- |
| Upregulated DEGs | NPTX2,NDUFA4L2,CA9,ANGPTL4,COL23A1,SLC6A3,KISS1R, APOC1, FABP7, NNMT, PNCK, CD70, CYP2J2, AHNAK2, LOX, INHBB, ENPP3, RGS1, CXCL9, IDO1, SCARB1, CP, EGLN3, IGFBP3, NETO2, FABP6, NKG7, ENO2, ANGPT2, SPAG4, GZMK, TMEM91, CCL5, ESM1, STC2, PNMA2, CDCA2, VWF, SCD, CXCL10, CXCR4, C1QB, GZMA, RNASET2, TNFAIP6, SIGLEC10, C3, SLC1A3, PLA2G7, CD2, ADM, CCL20, C1QC, CTHRC1, CD247, LAMA4, TYROBP, TMEM45A, TRIB3, TGFBI, PTHLH, CD300A |
| Downregulated DEGs | KNG1, CLCNKB, SERPINA5, SLC22A8, KCNJ1, TMEM213, SFRP1, SCNN1G, SLC12A3, XPNPEP2, ALDOB, TSPAN8, EGF, HPD, PLG, SH3GL2, GGT6, MAL, SCNN1A, DIO1, SLC34A1, DPEP1, TFCP2L1, ENPP6, ATP6V1B1, VTCN1, IYD, MT1G, TMPRSS2, ALB, HSD11B2, SLC12A1, C7, ACPP, PIGR, GPC5, SLC5A2, PROZ, SLC47A2, SCNN1B, TACSTD2, WNK4, DEFB1, MT1H, FAM151A, PPP1R1A, UPP2, DDN, PCK1, RAB25, SOSTDC1, IRX2, RHCG, PCSK1N, TMEM30B, ERP27, CLIC5, SLC4A1, SLC30A2, SLC22A7, ATP6V0A4, G6PC, GPC3 |
