# Supplementary material for: Identification of key genes CCL5, PLG, LOX and C3 in clear cell renal cell carcinoma through integrated bioinformatics analysis
Source: Front Mol Biosci. 2025 May 6;12:1587196. doi: 10.3389/fmolb.2025.1587196 (PMC12088980; doi:10.3389/fmolb.2025.1587196)
Supplement: Supplementary file 2 [file Supplementaryfile2.docx]

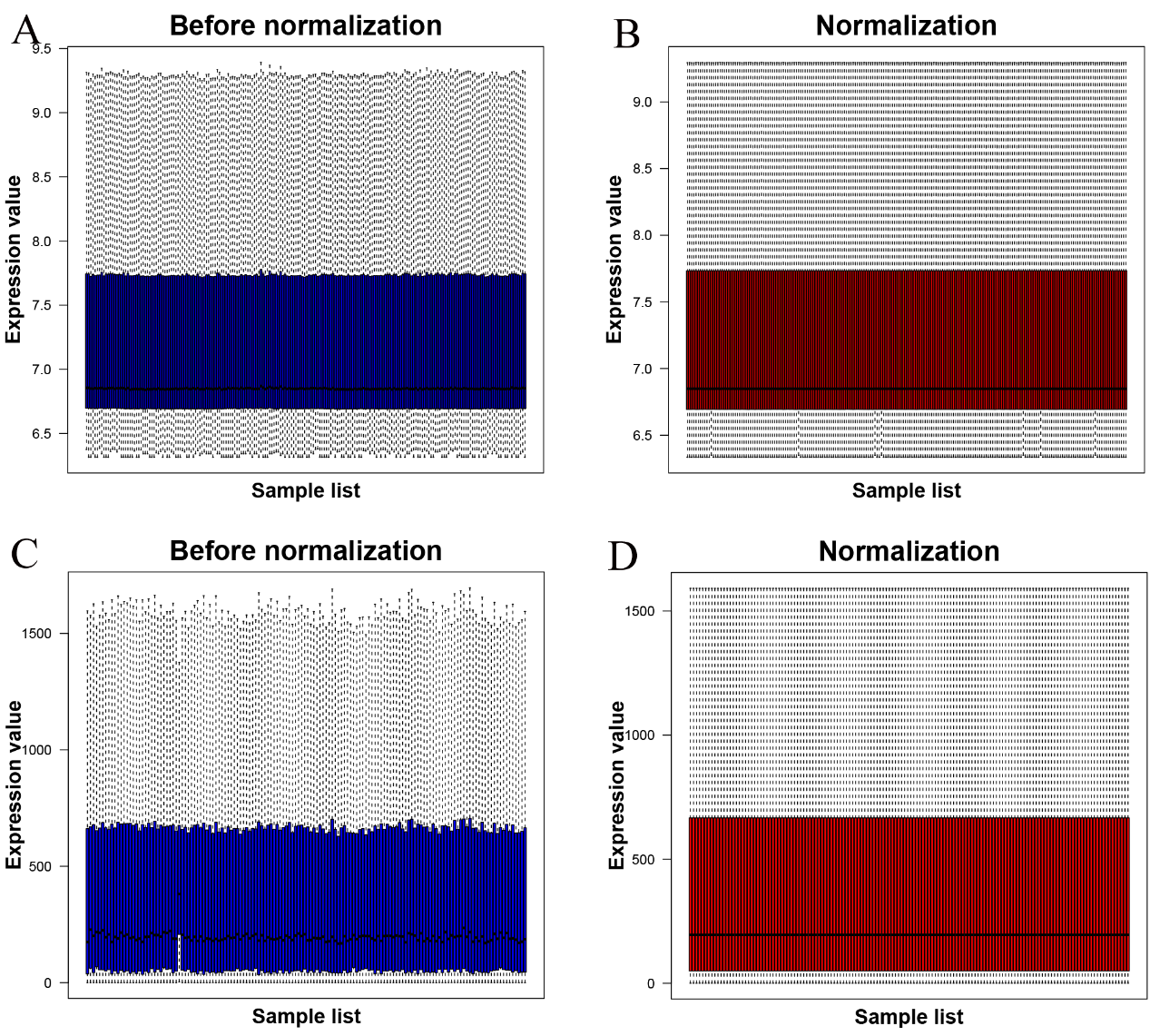


**Supplementary Figure 1. Standardization of gene expression by boxplot.**

(A) Gene expression profiles before standardization of GSE40435. (B) Gene expression profiles after standardization of GSE40435. (C) Gene expression profiles before standardization of GSE53757. (D) Gene expression profiles after standardization of GSE53757.


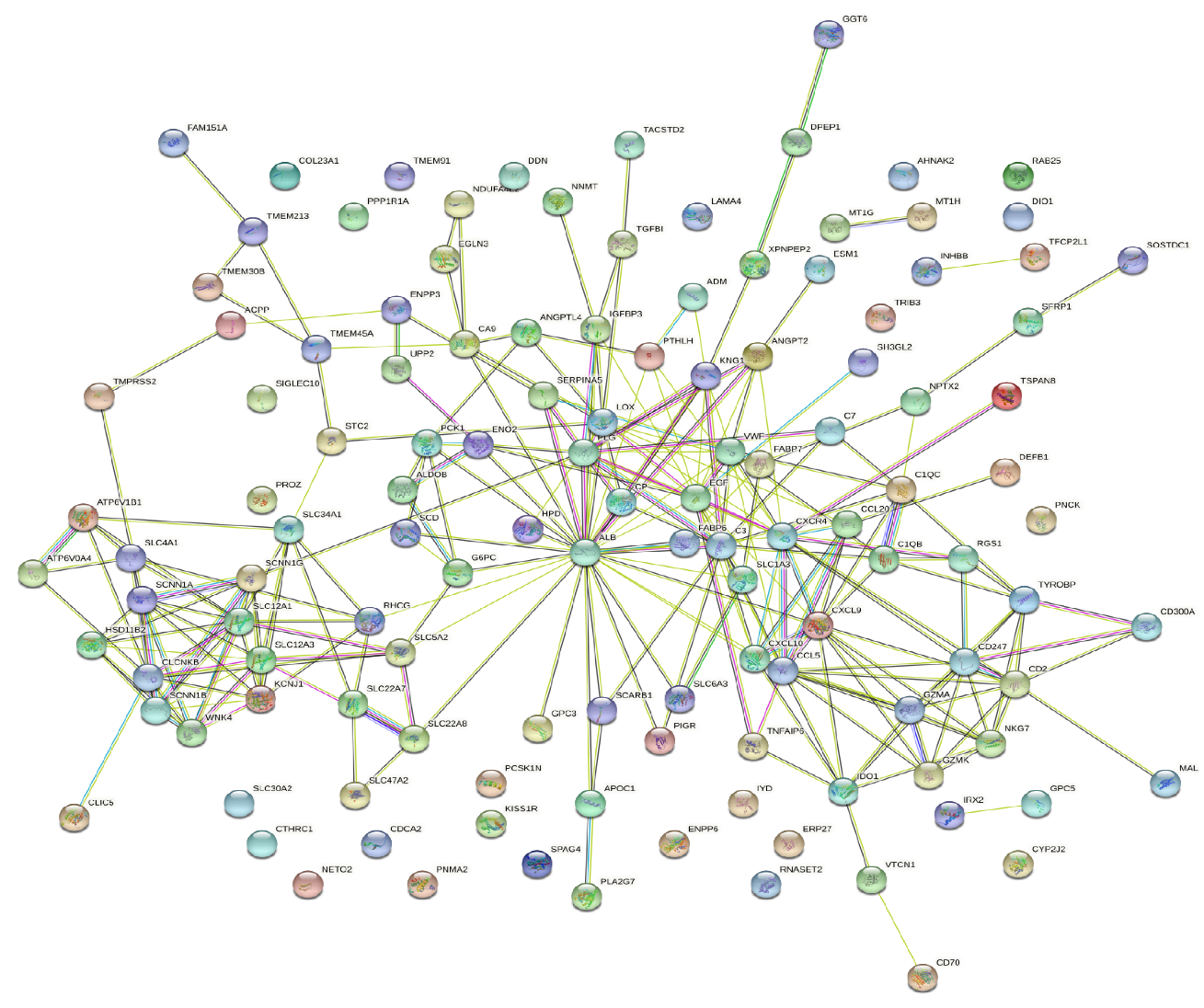


**Supplementary Figure 2. PPI network of DEGs.**


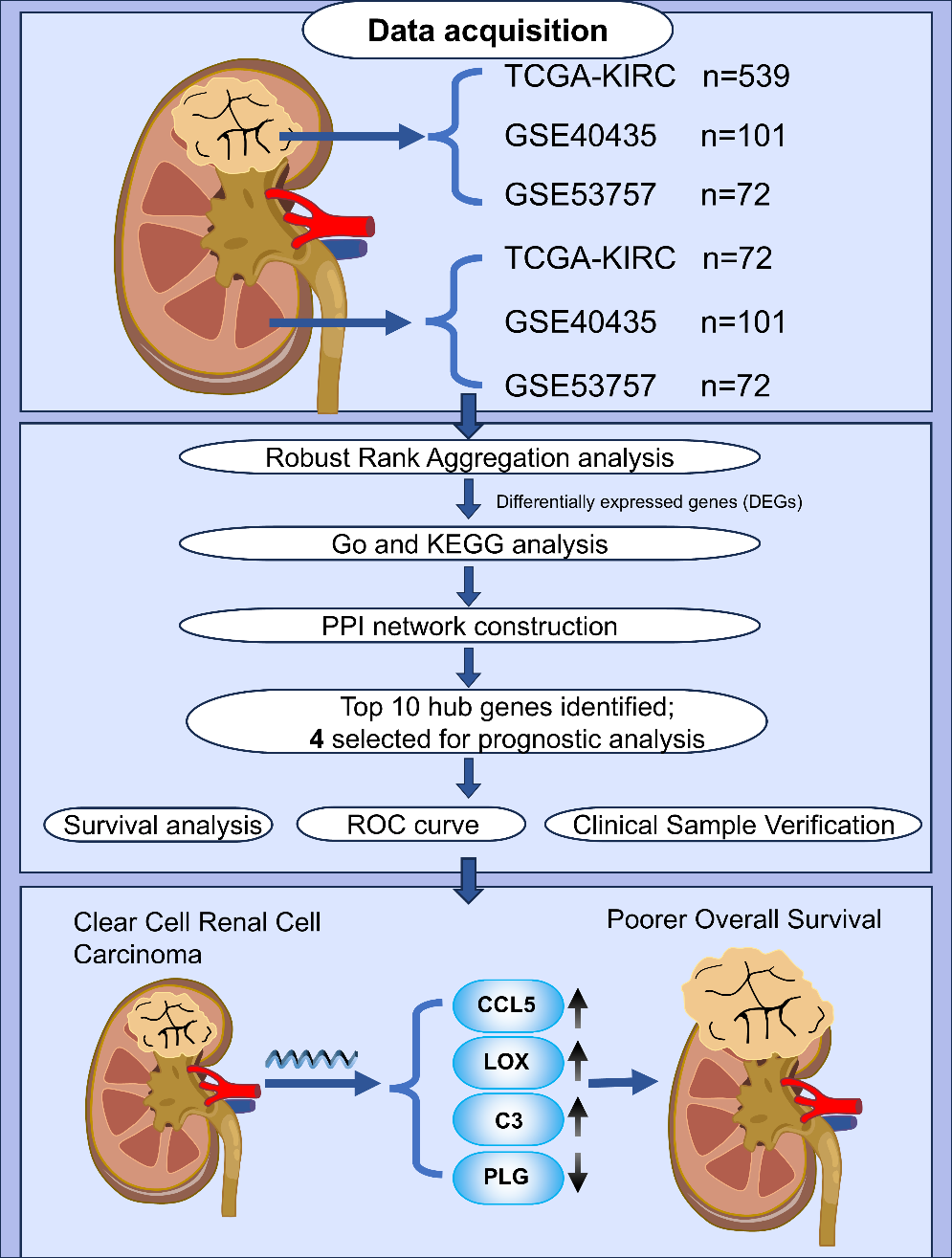


**Supplementary Figure 3.** **Graphical Abstract**
